# An exploratory, open-label, randomized, multicenter trial of hachimijiogan for mild Alzheimer’s disease

**DOI:** 10.3389/fphar.2022.991982

**Published:** 2022-10-14

**Authors:** Mosaburo Kainuma, Shinji Ouma, Shinobu Kawakatsu, Osamu Iritani, Ken-Ichiro Yamashita, Tomoyuki Ohara, Shigeki Hirano, Shiro Suda, Tadanori Hamano, Sotaro Hieda, Masaaki Yasui, Aoi Yoshiiwa, Seiji Shiota, Masaya Hironishi, Kenji Wada-Isoe, Daiki Sasabayashi, Sho Yamasaki, Masayuki Murata, Kouta Funakoshi, Kouji Hayashi, Norimichi Shirafuji, Hirohito Sasaki, Yoshinori Kajimoto, Yukiko Mori, Michio Suzuki, Hidefumi Ito, Kenjiro Ono, Yoshio Tsuboi

**Affiliations:** ^1^ Department of Japanese Oriental Medicine, Toyama University Hospital, Toyama, Japan; ^2^ Department of Neurology, School of Medicine, Fukuoka University, Fukuoka, Japan; ^3^ Department of Neuropsychiatry, Aizu Medical Center, Fukushima Medical University, Aizuwakamatsu, Japan; ^4^ Department of Geriatric Medicine, Kanazawa Medical University, Kanazawa, Ishikawa, Japan; ^5^ Translational Neuroscience Center, Graduate School of Medicine, International University of Health and Welfare, Tochigi, Japan; ^6^ Department of Neuropsychiatry, Graduate School of Medical Sciences, Kyushu University, Fukuoka, Japan; ^7^ Department of Neurology, Graduate School of Medicine, Chiba University, Chiba, Japan; ^8^ Department of Psychiatry, Jichi Medical University, Tochigi, Japan; ^9^ Division of Neurology, Second Department of Internal Medicine, Faculty of Medical Sciences, University of Fukui, Fukui, Japan; ^10^ Division of Neurology, Department of Medicine, Showa University School of Medicine, Tokyo, Japan; ^11^ Department of Neurology, Wakayama Medical University, Wakayama, Japan; ^12^ Department of General Medicine, Oita UniversityFaculty of Medicine, Oita, Japan; ^13^ Department of Internal Medicine, Wakayama Medical University Kihoku Hospital, Wakayama, Japan; ^14^ Department of Dementia Medicine, Kawasaki Medical School, Okayama, Japan; ^15^ Department of Neuropsychiatry, Graduate School of Medicine and Pharmaceutical Sciences, University of Toyama, Toyama, Japan; ^16^ Department of General Internal Medicine, Kyushu University Hospital, Fukuoka, Japan; ^17^ Center for Clinical and Translational Research, Kyushu University Hospital, Fukuoka, Japan; ^18^ Department of Rehabilitation, Fukui Health Science University, Fukui, Japan; ^19^ Department of Neurology, Kanazawa University Graduate School of Medical Sciences, Kanazawa, Ishikawa, Japan

**Keywords:** Alzheimer’s disease, hachimijiogan, ADAS‐Jcog, Kampo medicine, cognitive dysfunction

## Abstract

**Background:** Alzheimer’s disease (AD) is a progressive neurodegeneration and is the most prevalent form of dementia. Intervention at an early stage is imperative. Although three acetylcholinesterase inhibitors (AChEIs) are currently approved for the treatment of mild AD, they are not sufficiently effective. Novel treatments for mild AD are of utmost importance.

**Objective:** To assess the effectiveness of hachimijiogan (HJG), a traditional Japanese herbal medicine (Kampo), in the treatment of mild AD.

**Methods:** This exploratory, open-label, randomized, multicenter trial enrolled patients with mild AD whose score on the Mini Mental State Examination (MMSE) was over 21points. All participants had been taking the same dosage of AChEI for more than 3 months. The participants were randomly assigned to an HJG group taking HJG extract 7.5 g/day in addition to AChEI or to a control group treated only with AChEI. The primary outcome was the change from baseline to 6 months post treatment initiation on the Alzheimer’s Disease Assessment Scale-cognitive component- Japanese version(ADAS-Jcog). The secondary outcomes were change from baseline of the Instrumental Activity of Daily Life (IADL), Apathy scale, and Neuropsychiatric Inventory (NPI) -Q score.

**Results:** Among the 77 enrollees, the data of 69(34 HJG and 35 control)were available for analysis. The difference in the change of ADAS-Jcog from baseline to 6 months of the HJG and control groups was 1.29 (90% Confidence interval (CI), −0.74 to 3.32 *p* = 0.293). In the subgroup analysis, the differences in the change from baseline to 3 and 6 months for women were 3.70 (90% CI ,0.50 to 6.91, *p* = 0.059) and 2.90 (90% CI,0.09 to 5.71, *p* = 0.090), respectively. For patients over 65 years, the difference at 3 months was 2.35 (90%CI, 0.01 to 4.68 *p* = 0.099). No significant differences were found between the HJG and control groups in IADL score, Apathy scale, or NPI-Q score.

**Conclusion:** Although not conclusive, our data indicate that HJG has an adjuvant effect for acetylcholinesterase inhibitors and that it delays the deterioration of the cognitive dysfunction of mild Altzheimer’s disease patients.

**Clinical Trial Registration:** http://clinicaltrials.gov Japan Registry of clinical trials, identifier jRCTs 071190018

## Introduction

Cognitive and memory impairment in Dementia leads to negative changes in behavior and activities of daily life (Cipriani et al., 2020). More than 55 million people worldwide currently suffer from dementia, with an incidence of about 10 million each year. Estimates indicate it will rise to 78 million in 2030 and 139 million in 2050. Alzheimer’s disease (AD) is the most prevalent form of dementia, accounting for 60–70% of the disease (World health Organization, 2018).

Currently, AD is considered to start decades before clinical symptoms occur, and it is classified into three phases: preclinical AD, mild cognitive impairment (MCI) due to AD, and AD dementia (Sperling et al., 2011). Observational studies have reported associations between several vascular and lifestyle-related risk factors and increased risk of late-life cognitive impairment and AD, the most prevalent form of dementia (Norton et al., 2014). It is imperative that we focus on prevention of AD (prevention from the preclinical stage) and secondary prevention (intervention from MCI due to AD: early detection, early diagnosis, and early treatment) (McDade and Bateman., 2017). Numerous drug and non-drug therapies have been developed with the goal of alleviating or curing symptoms (Jeon et al., 2019). The currently recognized drugs are three acetylcholinesterase inhibitors (AChEIs) and one N-Methyl-D-Asparate(NMDA) receptor antagonist. Of note, NMDA receptor antagonist is not approved for mild AD. According to Tan, et al., AChEIs have benefits for stabilizing or slowing decline in cognitition, function, behavior and the clinical global change of patients with AD, however more dropouts and adverse events occurred with AChEIs compared to placebo (Tan et al., 2014) In contrast, previous studies reported no difference from placebo groups about 6 months after the initiation of AChEIs (Wolfson et al., 2002; Farlow et al., 2000; Winblad et al., 2006)^.^Recently, aducanumab, a new monoclonal antibody targeting aggregated beta-amyloid, was approved by the FDA. However, its clinical efficacy is uncertain because it is unclear whether or not reducing amyloid-βis clinically useful. Moreover, about 40% of the patients in clinical trials experienced vasogenic edema and cortical microhemorrhages, adverse events called amyloid-related imaging abnormalities. The initial price set by the manufacturer was prohibitively high (Rabinovici. 2021). Therefore, new therapeutic agents are essential.

Because traditional East Asian medicine has since ancient times been used to treat symptoms of what appears to be dementia, researchers are now turning to traditional medicines to identify potential neuroprotective or disease-modifying agents (Suzuki et al., 2005; Kaushik et al., 2018; Lim et al., 2018; Jeon et al., 2019; Watari et al., 2019). Traditional Chinese Medicine was introduced to Japan between the 5th and 6th centuries and has changed to suit Japanese needs over time to what is now called Kampo medicine. Kampo medicines have been approved by the Ministry of Health, Labor and Welfare of Japan for the treatment of the various diseases seen in specialty departments, and many doctors routinely prescribe Kampo medicine in clinical practice.

Hachimijiogan (HJG) is a traditional Japanese (herbal) medicine (Kampo) that is prescribed for the treatment of “kidney^[^™^]^ deficiency,” a key pathology of Kampo medicine. (Terasawa. 1993). It is effective for various symptoms common to the older population, such as low back pain, nocturia, numbness, and coldness in the legs. This study was done because we consider one of the mechanisms of cognitive impairment, from the Kampo medicine perspective, to be “kidney^[^™^]^ deficiency.” Hachimijiogan is composed of eight herbal components, for which five crude drugs (Rehmannia Root, Cornus Fruit, Dioscorea Rhizome, Moutan Bark and Cinnamon Bark) and their extracted ingredients have been reported to have efficacy against AD pathology (Jeon et al., 2019). There has only been one clinical study on HJG for dementia. According to Iwasaki,et al., HJG was effective for moderate to severe dementia (AD combined with cerebrovascular disease), but the results were not conclusive because of the small number of cases (Iwasaki et al., 2004). Our previous study demonstrated that HJG administration ameliorated memory impairment in rats treated with amyloid-βprotein and cerebral ischemia (Moriyama et al., 2017). Further, we found in a basic experiment that HJG has a neurotrophic effect and ameliorated the cognitive impairment of AD model rats *via* cAMP response element binding protein (CREB) activation (Kubota et al., 2017).

Based on our experience and our previous studies, this study was done to test our hypothesis that HJG would be safe and effective for the treatment of patients with mild AD.

## Materials and methods

### Study drug

We used TSUMURA hachimijiogan Extract Granules for Ethical Use (TJ-7, HJG, lot number P04531, Tokyo Japan). HJG is manufactured in compliance with Japanese good manufacturing practice (GMP) defined by Japanese law to ensure quality control. HJG is made by mixing the botanical drugs described below, followed by extraction using a hot water, and finally the extract is made into a powder by spray drying method. Its quality has been ascertained through quantification of the amounts of marker ingredients (paeoniflorin, loganin, benzoylmesaconine, and 14-anisoylaconine). The daily dose of HJG extract(7.5 g) includes dried botanical drugs in the following amounts: Japanese Pharmacopoeia[JP] Rehmannia Root(6.0 g), JP Cornus Fruit(3.0 g), JP Dioscorea Rhizome(3.0 g), JP Alisma Rhizome(3.0 g), JP Poria Sclerotium(3.0 g), JP Moutan Bark(2.5 g), JP Cinnamon Bark(1.0 g), and JP Powdered Processed Aconite Root(0.5 g) (The Ministry of Health, Labour, and Welfare, Japan, 2022). The detailed information on botanical drugs is provided in the Supplementary material. HJG was administered orally with warm water before a meal.

### Ethics

The study was conducted in accordance with the principles of the Declaration of Helsinki and Tokyo and approved by the Kyushu University Hospital Clinical Research Review Board (CRB) (Fukuoka, Japan) (CRB approval number: KD 2019001). We received written informed consent from all participants.

### Study design

This 24-week, open-label, randomized, multicenter trial was conducted at 14 sites in Japan between 2 August 2019 and 31 March 2022. We initially planned to recruit participants at three institutes according to the published protocol (Kainuma et al., 2020). However, because of the COVID-19 pandemic, the recruitment was delayed, so from January 2021 we were able to add 11 institutes. Our protocol was, in brief, that patients with mild AD were divided into an AChEI plus HJG group and an AChEI alone group to compare the effectiveness and safety of the addition of HJG. Patients diagnosed with AD were eligible if they met all the inclusion criteria. The main inclusion criteria were 1)age ≥50 to <85 years, 2) mild AD with a score on the Mini Mental State Examination(MMSE)of ≥21 points,3) taking the same dosage of AChEI (Donepezil, Galantamine, or Rivastigmine) for more than 3 months, but not taking NMDA receptor antagonist (Memantine). The exclusion criteria were as follows. 1) A change of medication dose that could affect the worsening of dementia during the past 3 months, 2) Kidney dysfunction (eGFR<30 ml/min/1.73 m^2^), 3) AST or ALT>100IU/L, 4) At least one complication with gastric ulcer, bronchial asthma, or epilepsy, 5) Taking Kampo Medicine other than HJG for more than the past 3 months, 6) patients determined to be ineligible for study by their doctors for reasons such as disease with serious complications.

### Outcome

The primary outcome was the change of the Alzheimer’s Disease Assessment Scale Cognitive Component- Japanese Version (ADAS-Jcog) from baseline to 6 months. The secondary outcome was the change from baseline to 6 months of the Instrumental Activity of Daily Life (IADL), the Apathy scale, and the Neuropsychiatric Inventory (NPI)-Q.

The safety outcomes included spontaneously reported adverse events (AEs) or serious AEs (SAEs).

### Randomization and masking

All participants met the eligibility criteria and gave written informed consent. Randomization was done with the Randomization Module of Research Electronical Data Capture (RED Cap). A computer-generated list of random numbers was transferred to RED Cap for block randomization by age and sex, then the participants were randomly assigned at a 1:1 ratio. Participants, physicians, and data evaluators were aware of the allocation group after randomization, but outcome evaluators were blinded.

### Procedure

After randomization, the HJG group took 2.5 g of HJG extract 3 times/day in addition to AChEI for 6 months and the control group continued to take only AChEI. Patients underwent assessments at baseline and after 3 and 6 months.

AEs, or SAEs, vital signs (temperature, heart rate, and blood pressure), and physical examination and laboratory tests were evaluated each visit.

### Statistical analysis

SAS^®^ software (version 9.4; SAS Institute Japan Ltd., Tokyo, Japan) was used for all statistical analyses.

An efficacy analysis was done on the per protocol sets (PPS), with the same analysis of the full analysis sets (FAS) done for sensitivity analysis to confirm the accuracy of the results. From all subjects, FAS will exclude cases of serious noncompliance with clinical research methods, cases not administered, and cases not observed (complete absence of the primary outcome). A group consisting of subjects who are included in the FAS and do not meet the following conditions is defined as a PPS. 1) Subjects who do not meet the selection criteria specified in the protocol or who violate the exclusion criteria. 2) Significant deviations from the study protocol. 3) Subjects who failed to receive adequate doses of the study drug (medication compliance rate <75%). To analyze the primary outcome, an analysis of covariance was done for the change in ADAS-Jcog from baseline for the PPS, with treatment group and baseline values as covariates. Continuous variables are expressed as the mean value and standard deviation. The analysis of secondary outcomes was also done in the same way as the analysis of the primary outcome of the PPS. For statistical analyses, a two-tailed test at the 10% level of significance was done. Two-tailed interval estimation was done with a 90% confidence interval(CI) and corresponding *p* values (with significance defined as *p* < 0.10).

The data of any participant who received even one medication was eligible for the Safety Analysis Set (SAS) evaluation, with the number and incidence of liver dysfunction, interstitial pneumonia, and gastrointestinal disorders calculated for both groups.

## Results

The first patient was enrolled on 5 September 2019 and the last patient completed the study on 24 March 2022: 77 patients were enrolled and randomly assigned.

**FIGURE 1:**
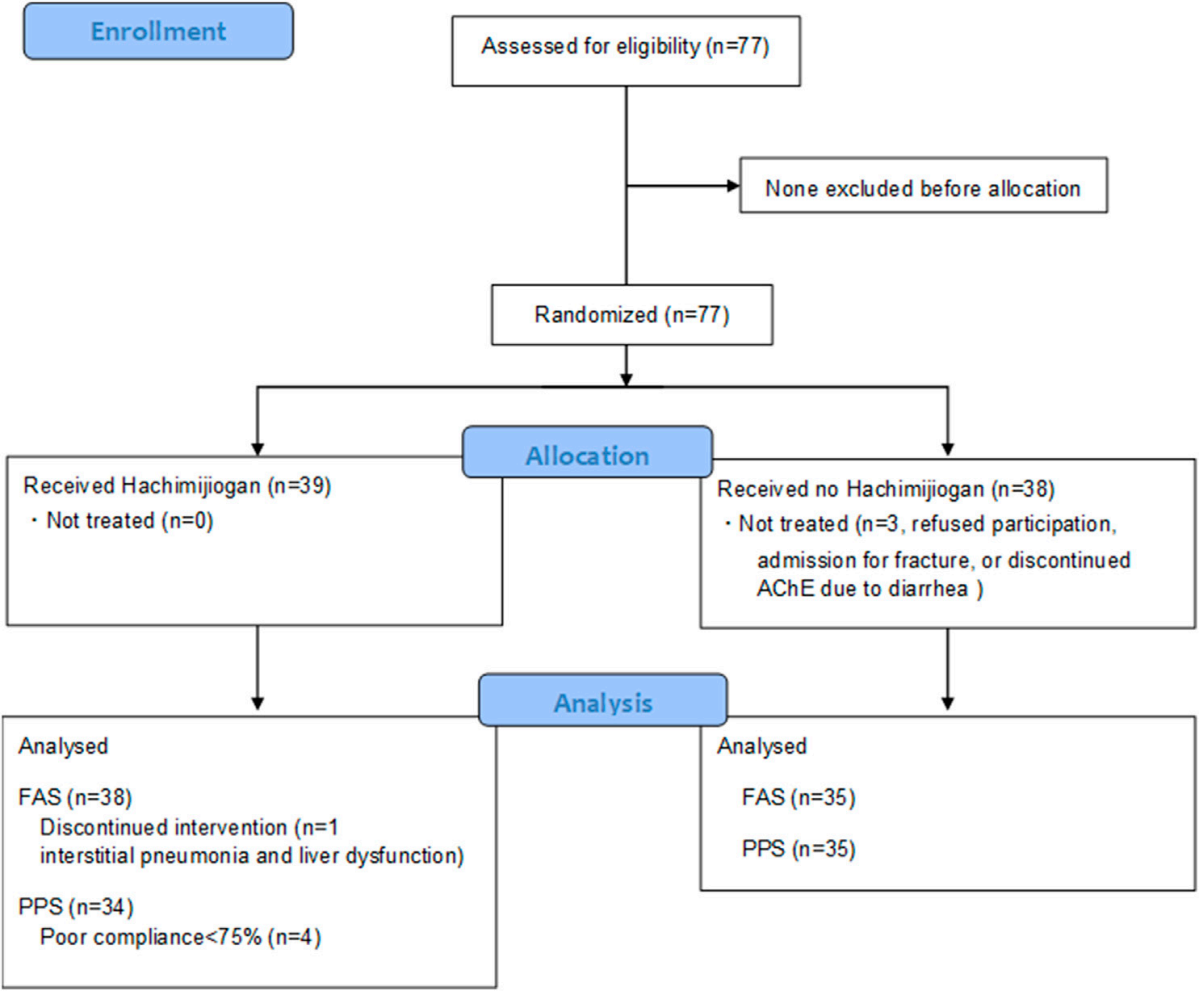
Enrollment, Randomization, and Trial completion.

**FIGURE 2:**
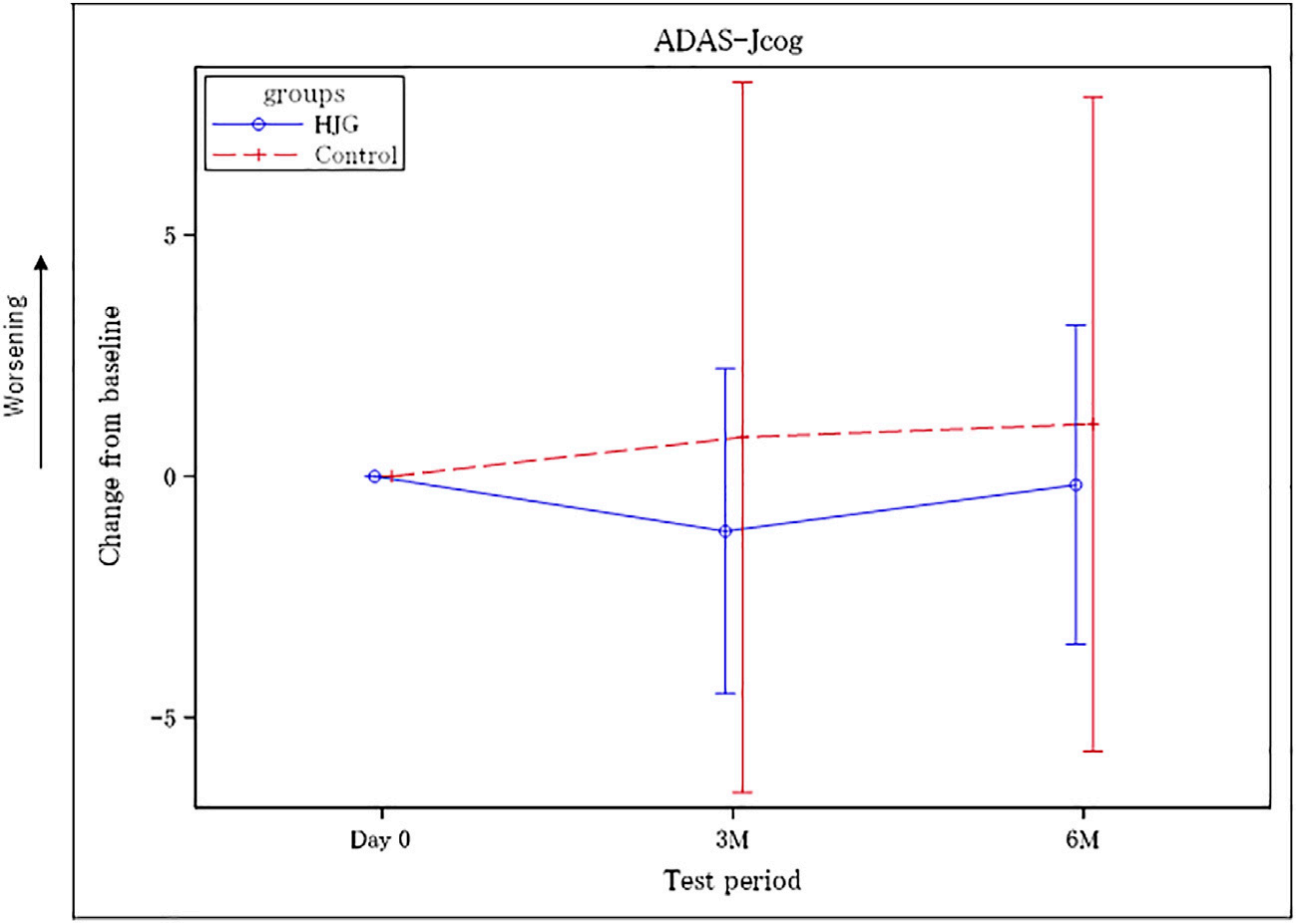
Difference between the HJG and control groups in the change from baseline to 3 and 6 months: ADAS-Jcog.

**FIGURE 3:**
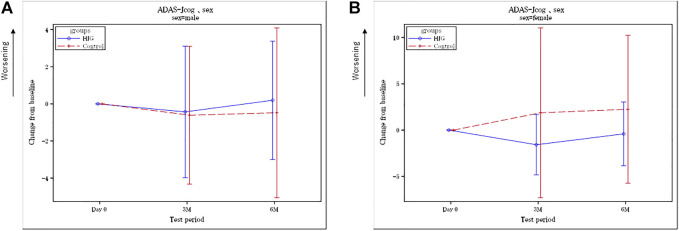
**(A)** Difference between the HJG and control groups in the change from baseline to 3 and 6 months: ADAS-Jcog for male participants. **(B)** Difference between the HJG and control groups in the change from baseline to 3 and 6 months: ADAS-Jcog for female participants.

No difference was found in the background factors of the HJG and Control groups (Table 1). From among the 77 enrollees, the data of 69(34 HJG and 35 control)were available for analysis(Figure 1). In the analysis of ADAS-Jcog, the difference in the change from baseline at 6 months of the HJG and control groups was 1.29 (90%CI, −0.74 to 3.32 *p* = 0.293). The difference at 3 months was 1.98 (90% CI, −0.17 to 4.14 *p* = 0.130) (Figure 2). Furthermore, in the subgroup analysis by sex, the differences from baseline to 3 and 6 months for male participants were -0.23 (90%CI, −2.61 to 2.16 *p* = 0.873) and −0.66 (90%CI, −3.30 to 1.97 *p* = 0.671), respectively (Figure 3A).

**TABLE 1:** Baseline characteristics.

	HJG group(*n* = 39)	Control group(*n* = 35)
Sex(male/female)	15/24	15/20
Age (years)	75 ± 6.6 (73–77)	76 ± 7.1 (74–78)
Height (cm)	157 ± 9.2 (154–160)	157 ± 7.8 (155–160)
Body weight (kg)	55 ± 11 (52–59)	54 ± 11 (50–57)
Body mass index (kg/m^2^)	22 ± 3.5 (21–23)	22 ± 3.2 (20–23)
BUN (mg/dL)	16 ± 4.3 (15–18)	17 ± 5.2 (16–19)
Creatinine (mg/dL)	0.80 ± 0.21 (0.73–0.87)	0.79 ± 0.26 (0.70–0.88)
eGFR (mL/min/1.73m^2^)	64 ± 17(58–69)	67 ± 18 (60–73)
Albumin (g/dL)	4.1 ± 0.3 (4.0–4.2)	4.1 ± 0.3 (4.1–4.2)
HbA1c (%)	6.0 ± 1.0 (5.7–6.3)	6.1 ± 1.0 (5.8–6.4)
Systolic Hypertension (mmHg)	131 ± 18 (126–137)	132 ± 17 (127–138)
Diastolic Hypertension (mmHg)	76 ± 13 (72–80)	79 ± 11 (75–82)
Disease duration, years	3.6 ± 2.8 (2.7–4.5)	2.7 ± 2.2(2.0–3.4)
Education (university/non university)	12/27	6/29
ADS-Jcog	14 ± 5.2 (13–16)	14 ± 5.6(13–16)
IADL	5.2 ± 2.5 (4.4–6.0)	4.9 ± 2.2(4.1–5.7)
NPI-Q score	5.6 ± 7.7 (3.1–8.1)	4.4 ± 8.5(1.5–7.3)
Apathy scale	14 ± 7.0 (11–16)	14 ± 6.5(12–17)
Hypertension, n(%)	15(39%)	17(45%)
Dyslipidemia, n(%)	11(28%)	13(36%)
Diabetes mellitus, n(%)	7(18%)	6(16%)
Old brain infarction, n(%)	1(3%)	0(0%)
AChE inhibitor		
Donepezil	20 (51%)	24 (69%)
Galantamine	11 (28%)	7 (20%)
Rivastigmine	8 (21%)	4 (11%)

**FIGURE 4:**
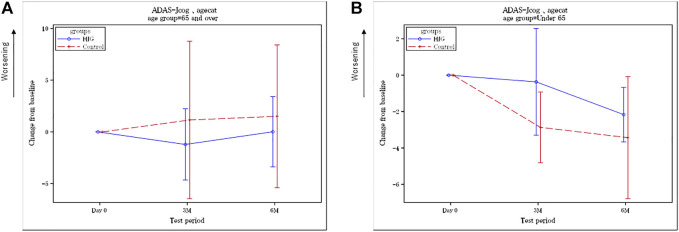
**(A)** Difference between the HJG and control groups in the change from baseline to 3 and 6 months: ADAS-Jcog for participants over 65 years. **(B)** Difference between the HJG and control groups in the change from baseline to 3 and 6 months: ADAS-Jcog for participants under 65 years.

In contrast, the differences in change from baseline to 3 and 6 months for female participants were 3.70 (90%CI, 0.50 to 6.91 *p* = 0.059) and 2.90 (90%CI, 0.09 to 5.71, *p* = 0.090), respectively (Figure 3B). For age, the differences in the change from baseline to 3 and 6 months for participants ≥65 years were 2.35 (90%CI, 0.01 to 4.68 *p* = 0.099) and 1.47 (90%CI, −0.71 to 3.66, *p* = 0.265), respectively (Figure 4A), and the differences for participants <65 years were −2.18 (90%CI, −6.35 to 1.99 *p* = 0.307) and −0.79 (90%CI,-2.82 to 1.24, *p* = 0.427), respectively (Figure 4B). No significant difference in education was found.

In the analysis of IADL, the differences between the HJG and control groups in the change from baseline to 3 and 6 months for male participants were 0.33 (90%CI, −0.07 to 0.72 *p* = 0.170) and 0.38 (90%CI,-0.31 to 1.08 *p* = 0.352), respectively. For the female participants they were 0.48 (90%CI, −0.10 to 1.07 *p* = 0.168) and 0.37 (90%CI,-0.40 to 1.15 *p* = 0.425), respectively.

In the analysis of the apathy scale, the differences between the HJG and control groups in the change from baseline to 3 and 6 months were 1.46 (90%CI, −0.67 to 3.58 *p* = 0.256) and 0.19 (90% CI, −1.60 to 1.99 *p* = 0.858), respectively.

In the analysis of NPI-Q, the differences between the HJG and control groups in the change from baseline to 3 and 6 months were-1.51 (90%CI, −4.01 to 0.99 *p* = 0.317) and −0.89 (90%CI, −3.07 to 1.29 *p* = 0.497), respectively. With FAS, the same results were gained for the primary and secondary points. In the HJG group, only one participant discontinued, because of interstitial pneumonia and liver dysfunction.

## Discussion

This exploratory study was the first to investigate the effectiveness and safety of adding HJG to AChEI for the treatment of the cognitive impairment of patients with mild AD.

Our results (Figure 2) showed that the ADAS-Jcog worsened over time in the control group, in which the participants continued to receive AChEI alone, whereas the group that received HJG had delayed cognitive deterioration for 6 months, although the difference was not significant. Analysis by sex showed that the women in the HJG group showed more significant improvement in cognitive function at both 3 and 6 months than the women in the control group who received AChEI alone. (Figure 3B). For the participants over 65 years, at 3 months a more significant improvement in cognitive function was seen in the HJG group compared with the control group (Figure 4A). HJG has proven effective for various age-related diseases, and our results showing effectiveness for participants over 65 years-of-age indicate that cognitive dysfunction may be a symptom of “kidney deficiency.” Our data support HJG as being effective for older patients with mild cognitive dysfunction.

In our analysis of the ADAS-Jcog as a whole and of women only, the HJG group tended to have improvement in cognitive function at 3 months, but deterioration was observed at 6 months. AChEI has been reported to improve cognitive function after 3–6 months of treatment, but a decline was seen after 9 months of treatment (Farlow M et al., 2000; Winblad B et al., 2006). Nine patients (2 HJG, 7 Control) in our study were enrolled immediately after AChEI for 3 months, which may have resulted in a synergistic effect of HJG and AChEI at the 3 months point. However, at 6 months, 9 months after the start of AChEI administration, the effect would be that of HJG alone. To examine the hypothesis, we did a post-hoc statistical analysis, and the difference in the change of ADAS-Jcog from baseline to 3 and 6 months of the HJG and control groups was 2.25 (90% CI, −0.19 to 4.70 *p* = 0.129), and 0.87 (90% CI, −1.31 to 3.04 *p* = 0.509), respectively. From these results, excepting the patients recruited immediately after 3 months administration of AChEI, the group difference was increased at 3 months, although the difference was not significant.

The present study design included three points of change in ADAS-Jcog as the minimum clinically important differences (MCID), in reference to previous studies (Department of Health and Human Services et al., 1989; Schrag et al., 2012), but no clinical study of aducanumab or other antibody drugs that have been done in recent years on patients with mild AD have shown MCID in ADAS-Jcog exceeding 2 points (Doody et al., 2014; Honig et al., 2018; US Food and Drug Administration., 2020; Mintun et al., 2021), and no consensus has been reached on MCID in ADAS-Jcog (Rockwood et al., 2010; Liu et al., 2021). We also set the MCID at 3 points, but this number is the minimum amount of change for an individual subject and may have been overstated for the MCID of the ADAS-Jcog in our participants with mild AD. However, the difference between the two groups in the study of women was 2.90 at 6 months. Scltzer B et al. reported a group difference on ADAS-cog of 2.3 at 24 weeks for donepezil and placebo groups with Early-stage AD (Seltzer et al., 2004). Further, a solanezumab trial of mild dementia due to AD reported a difference of −0.80 at 80 weeks (Honig et al., 2018). With donanemab for early AD the difference was -1.86 at 76 weeks (Mintun et al., 2021). Although the primary outcome point of these two studies was over 24 weeks, the figures of these two studies show group differences that were obviously lower than ours. These data suggest that HJG may have been related to the improvement of cognitive function when compared to the rate of change in recent clinical studies on mild AD.

Only one patient in the HJG group had to drop out of the study, because of interstitial pneumonia. It is important to note that this patient had lung disease at the start of the study. This indicates that risk from the administration of HJG is very low and that it is safe to use. Although our results were not conclusive, they do show that HJG is safe and effective for clinical use and that its clinical use would benefit patients with mild AD.

The present study also showed that AChEI was effective for some male patients. The patients in the final PPS analysis of the HJG group included 21 women and 13 men, resulting in a lack of statistical power and a statistically insignificant difference. In our protocol, the eligibility criteria were set based on the assumption that fixing the AChEI dose for more than 3 months would reduce the number of dropouts due to side effects caused by AChEI. In future study it will be important to verify the efficacy of HJG by examining a larger number of patients for more than 6 months after taking AChEIs.

The Apathy Score is used to assess motivation in the cerebrovascular disease sequelae, but it is well known that, with regard to AD, depression may be present or it may be optimistic with no disease awareness in its early stages. No significant difference was found in a donepezil group compared to controls in a study of early AD that used the Apathy scale (Seltzer et al., 2004)

Donepezil has been reported to improve the instrumental and basic ADLs of moderate to severe AD patients (Feldman et al., 2006). However, we could not find reports in which Donepezil was effective for early AD. According to previous studies, as the severity of AD progresses, ADL decreases and the burden on caregivers increases (Sun et al., 2018; Kawaharada et al., 2019). The severity of behavioral disorders and cognitive, psychological, and motor impairment of patients with AD have been positively correlated with increasing levels of caregiver burden of distress (Raggi et al., 2015). In this study, we used the NPI-Q score to assess caregiver burden; however, it was done with mild AD patients, and no difference between the groups was found in apathy score or IADL, which would indicate no difference in the burden of caregiving.

There are several limitations in the present study. The sample size available for analysis was small, especially that of men. Also, it was not placebo-controlled and was done over a relatively short-term period. Studies including a long-term treatment protocol and better-balanced samples will be necessary to verify the current results.

## Conclusion

Although our data are not conclusive, they indicate that HJG has an adjuvant effect for acetylcholinesterase inhibitors and that it is effective for delaying the cognitive dysfunction commonly seen in mild Alzheimer’s disease patients.

## Data Availability

The original contributions presented in the study are included in the article/Supplementary Material, further inquiries can be directed to the corresponding author.

## Abbreviations

AChEI, acetylcholinesterase inhibitor; AD, Alzheimer’s disease; ADAS-Jcog, Alzheimer’s disease assessment Scale-cognitive component-Japanese version; AEs, all adverse events; CREB, cAMP response element binding protein; FAS, full analysis set; HJG, Hachimijiogan; IADL, instrumental activity of daily Life; MCID, minimally-clinically important difference; MMSE, mini mental state Examination; NPI, nueropsychiatric inventory; PPS, per protocol set; PS, RED Cap, Research electronical data capture; SAS, safety analysis set.
